# Development of an algorithm to automatically compress a CT image to visually lossless threshold

**DOI:** 10.1186/s12880-017-0244-2

**Published:** 2018-12-17

**Authors:** Chang-Mo Nam, Kyong Joon Lee, Yousun Ko, Kil Joong Kim, Bohyoung Kim, Kyoung Ho Lee

**Affiliations:** 1 Department of Radiology, Seoul National University Bundang Hospital, Seoul National University College of Medicine, 82 Gumi-ro 173 Beon-gil, Bundang-gu, Seongnam-si, Gyeonggi-do 13620 Korea; 2 Division of Biomedical Engineering, Hankuk University of Foreign Studies, Oedae-ro 81, Mohyeon-myeon, Cheoin-gu, Yongin-si, Gyeonggi-do 17035 Korea

**Keywords:** Visually lossless threshold, CT compression, DICOM header

## Abstract

**Background:**

To develop an algorithm to predict the visually lossless thresholds (VLTs) of CT images solely using the original images by exploiting the image features and DICOM header information for JPEG2000 compression and to evaluate the algorithm in comparison with pre-existing image fidelity metrics.

**Methods:**

Five radiologists independently determined the VLT for 206 body CT images for JPEG2000 compression using QUEST procedure. The images were divided into training (*n* = 103) and testing (*n* = 103) sets. Using the training set, a multiple linear regression (MLR) model was constructed regarding the image features and DICOM header information as independent variables and regarding the VLTs determined with median value of the radiologists’ responses (*VLT*_*rad*_) as dependent variable, after determining an optimal subset of independent variables by backward stepwise selection in a cross-validation scheme.

The performance was evaluated on the testing set by measuring absolute differences and intra-class correlation (ICC) coefficient between the *VLT*_*rad*_ and the VLTs predicted by the model (*VLT*_*model*_). The performance of the model was also compared two metrics, peak signal-to-noise ratio (PSNR) and high-dynamic range visual difference predictor (HDRVDP). The time for computing VLTs between MLR model, PSNR, and HDRVDP were compared using the repeated ANOVA with a post-hoc analysis. *P* < 0.05 was considered to indicate a statistically significant difference.

**Results:**

The means of absolute differences with the *VLT*_*rad*_ were 0.58 (95% CI, 0.48, 0.67), 0.73 (0.61, 0.85), and 0.68 (0.58, 0.79), for the MLR model, PSNR, and HDRVDP, respectively, showing significant difference between them (*p* < 0.01). The ICC coefficients of MLR model, PSNR, and HDRVDP were 0.88 (95% CI, 0.81, 0.95), 0.85 (0.79, 0.91), and 0.84 (0.77, 0.91). The computing times for calculating VLT per image were 1.5 ± 0.1 s, 3.9 ± 0.3 s, and 68.2 ± 1.4 s, for MLR metric, PSNR, and HDRVDP, respectively.

**Conclusions:**

The proposed MLR model directly predicting the VLT of a given CT image showed competitive performance to those of image fidelity metrics with less computational expenses. The model would be promising to be used for adaptive compression of CT images.

## Background

Although the cost of storage and network resources have continued to drop, there is still a demand for irreversible compression of computed tomography (CT) images for long-term preservation and efficient transmission of data, especially between institutions at the regional or national level [1–4]. However, the irreversible compression is not always accepted by radiologists due to concern about compression artifacts that might hinder diagnosis. Therefore, the importance of achieving an optimal compression level for a CT image, which provides the maximum data reduction while preserving the diagnostic accuracy, has gained the attention of radiologists [5].

Regarding the estimation of such optimal compression level, many researchers have advocated that visually lossless threshold (VLT) is robust and conservative sufficiently to be adopted for the compression of medical images [6–11]. This approach focuses on image fidelity (i.e. the visual equivalence between the original and compressed images). The underlying idea of this approach is that if a compressed image is visually indistinguishable from its original, the artifacts should not affect the diagnosis.

However, because the compressibility of an image is affected by various factors including body parts, scanning protocols, and image contents itself, the establishment of a robust VLT for various images would require a very large study [12, 13]. Instead, if a computerized algorithm can accurately predict visual perception of radiologists, it can be used for compressing a CT image adaptively and automatically to its own VLT. For that purpose, several researchers have experimented with using image fidelity metrics, which measure the fidelity of a distorted image, in predicting the VLT, and some of the metrics showed promising results [5, 14–18]. However, to achieve the VLT of a given CT image, it is necessary to iteratively compress the image to multiple compression levels and to measure the image fidelity at each compression level until the measured fidelity reaches a cutoff value predefined by the metric.

It has been advocated that some of the factors influencing the compressibility of CT images can be derived directly from the Digital Imaging and Communications in Medicine (DICOM) header information [19, 20], especially related to the image noise which is known as an important factor affecting the compressibility of CT images as well as from image contents themselves [21–25]. Thereby, it is plausible to hypothesize that the VLTs of CT images can be predicted by using those DICOM header information and image features derived from the original images.

This study aimed to develop a computerized algorithm to predict the VLTs of CT images solely using the original images by exploiting the image features and DICOM header information for Joint Photographic Experts Group 2000 (JPEG2000) compression and to evaluate the performance of the algorithm in comparison with pre-existing image fidelity metrics.

## Methods

**Fig. 1:**
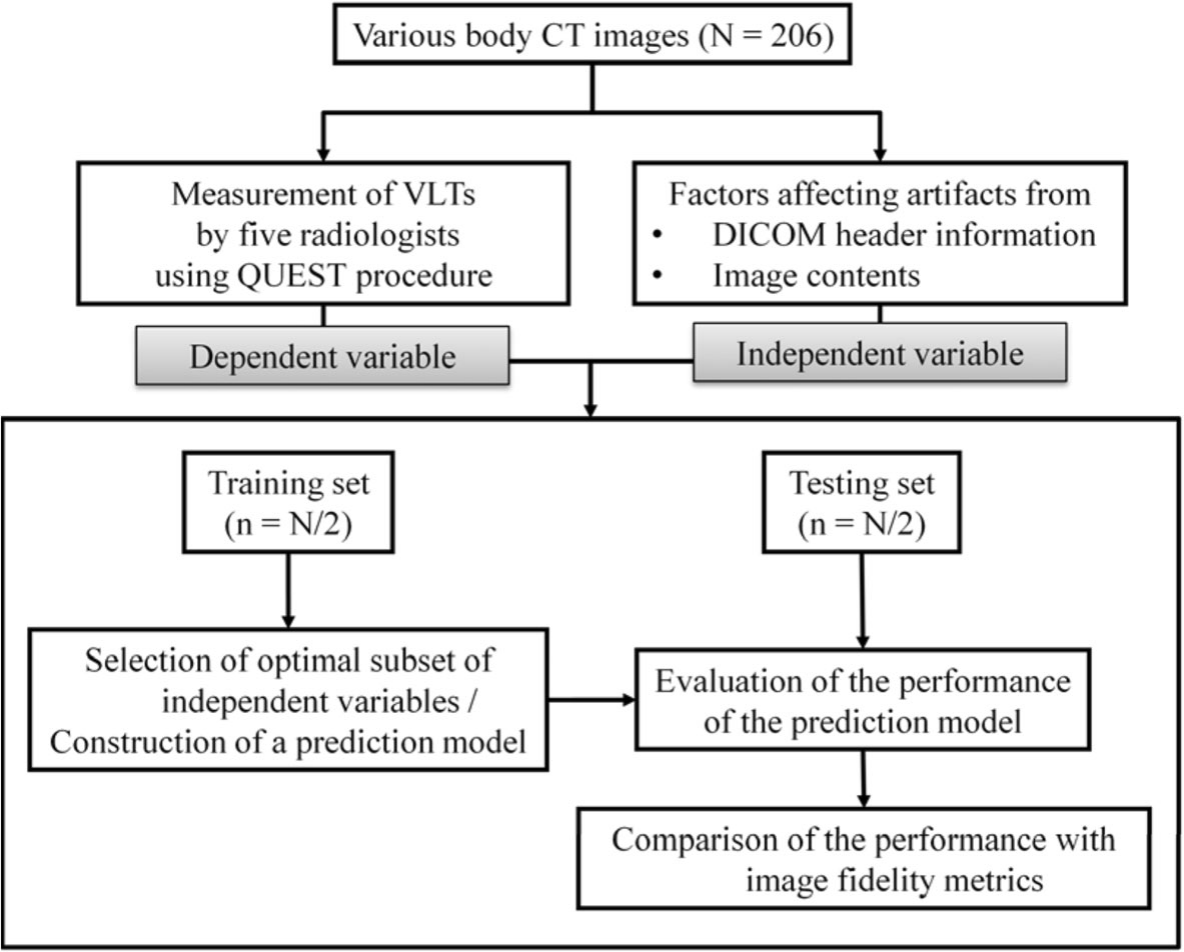
Study design. ^*^Independent variables include five image features (image standard deviation, image entropy, relative percentage of low frequency energy, variation in high frequency, and visual complexity) and DICOM header information (effective mAs, field of view, section thickness, and reconstruction filter). DICOM: digital imaging and communications in medicine; VLT: visually lossless threshold

Our institutional review board approved this study and waived informed patient consent. The study design was described in Fig. 1.

### A. Image acquisition and selection

A body radiologist with 13 years of clinical experience, who did not participate in the human visual analysis, retrospectively reviewed CT scans of adults which were obtained from 256-channel or 64-channel multi-detector row CT scanners (Brilliance; Philips Medical Systems, Cleveland, OH) in Seoul National University Bundang Hospital in early 2012. He compiled 206 studies (84 abdomen scans, 82 chest scans obtained by using our standard radiation dose, and 40 low-dose chest scans; 79 scans from 256-channel scanners and 127 scans from 64-channel scanners) containing common abnormalities.

Of the 84 abdomen scans, 42 scans were randomly selected and then reconstructed into 4-mm-thick transverse sections and the remaining 42 scans were reconstructed into 2-mm-thick sections. Likewise, 41 of the 82 standard-dose chest scans were reconstructed into 3-mm-thick sections and the remaining 41 scans were reconstructed into 2-mm-thick sections. The 40 low-dose chest scans were reconstructed into 3-mm-thick sections. From each reconstructed image dataset, the same radiologist selected a single section that most clearly represented pathology. Therefore, the final study sample was a set of 206 images, which was composed of five subsets of different body regions (i.e., abdomen or chest) and image noise levels (i.e., different section thicknesses and radiation doses): 4-mm-thick abdomen images (abdomen thick), 2-mm-thick abdomen images (abdomen thin), 3-mm-thick lung images (chest thick), 2-mm-thick chest images (chest thin), and 3-mm-thick low-dose chest CT images (low-dose chest).

Among various clinical CT protocols, we focused on these five CT protocols because they are performed very frequently in daily practice of our hospital and thus cover a large portion of the image archive. The scan parameters and patient demographics for each subset are tabulated in Table 1. All scan parameters followed the clinical scan protocols of our hospital. Also, using the five different subsets was to introduce heterogeneity in the test dataset to some extent in terms of structural content and image noise. The heterogeneity was considered important in measuring the robustness of the prediction model in predicting the VLTs of CT images, as it is well known that the image compressibility is significantly affected by structural content [17, 26] and image noise level [12, 13].

**Table 1:** CT imaging parameters and patient demographics

	Subsets				
	Abdomen thick	Abdomen thin	Chest thick	Chest thin	Low-dose chest
Common scan parameters	Detector collimation, 64 × 0.625 mm for 64-channel MDCT and 2 × 128 × 0.625 mm for 256-channel MDCT; gantry rotation time, 0.42 s for 64-channel MDCT and 0.27 s for 256-channel MDCT; tube potential, 120 kVp; pitch, 1.077 to 1.172; matrix, 512 × 512				
Body part	Abdomen	Abdomen	Chest	Chest	Chest
Field of view^ab^	277.2 ± 20.7 (245–323)	281.0 ± 23.5 (249–321)	319.0 ± 27.1 (262–383)	317.9 ± 26.5 (271–375)	291.5 ± 22.9 (248–329)
Section thickness (mm)	4	2	3	2	3
Effective mAs^ac^	121.5 ± 38.0 (69–222)	127.5 ± 34.2 (62–191)	152.3 ± 45.1 (58–249)	151.1 ± 30.5 (72–181)	25.6 ± 2.3 (22–31)
Effective radiation Dose (mSv)^ad^	7.5 ± 1.0 (4.5–9.8)	7.5 ± 1.5 (4.1–10.2)	7.2 ± 1.1 (4.3–9.5)	7.3 ± 0.9 (4.1–9.3)	1.6 ± 0.1 (1.4–1.7)
Reconstruction filter	Soft-tissue	Soft-tissue	Medium-sharp	Medium-sharp	Medium-sharp
Age^a^	57.2 ± 27.2 (15–88)	55.3 ± 24.8 (15:82)	50.6 ± 26.4 (18–88)	53.1 ± 24.4 (22–91)	45.4 ± 22.4 (19–84)
Sex (Male:Female)	22:20	23:19	22:19	20:21	23:17

Note: ^a^Data are means ± standard deviations, with ranges in parentheses. ^b^The field of view was set for each patient to match the maximum transverse diameter of the body to the image size. ^c^Automatic tube-current modulation was used. ^d^Estimated by multiplying the dose-length product measured on the CT console by a conversion factor (0.017 and 0.019 mSv•mGy-1•cm-1 for abdomen and chest, respectively) [39]

### B. VLT measurement by radiologists using QUEST procedure

Five radiologists with 10, 9, 7, 6, and 4 years of clinical experience, respectively, participated in the VLT measurement. The 206 images were randomly assigned to five reading sessions. The order of the reading sessions was changed for each reader. Sessions were separated by a minimum of 24 h to minimize reader fatigue.

Images were displayed in a monochrome monitor calibrated according to the Digital Imaging and Communications in Medicine part 14 grayscale standard display function [26]. Detailed specifications of the display system and viewing condition were described in Table 2. The window level and width were set as 20 and 400 HU for abdomen CT images and as −600 and 1500 HU for lung CT images, which were the default window settings in our clinical practice.

**Table 2:** Display system and viewing conditions in human visual analysis

Display system	
Display resolution	1536 × 2048 pixels
Display size	31.8 × 42.3 cm
Image resolution	1483 × 1483 pixels (stretched using bilinear interpolation)
Luminance	1.5–408.2 cd/m^2^
Viewing conditions	
Ambient room light	30 lux
Reading distance	42–77 cm
Window setting	level, 20 HU; width, 400 HU for abdomen CT,
	level, −600 HU; width, 1500 HU for chest CT, not adjustable
Magnification	not allowed
Reading time	not constrained

The five readers independently measured the VLT of each image by visually comparing the image with its distorted versions compressed to various compression ratios (CRs) using the JPEG2000 algorithm (Accusoft-Pegasus Imaging, Tampa, Fla) [27–30]. The VLT of each image was determined through 25 comparison trials. In each trial, the image pair of the original and compressed version was alternately displayed on a single monitor. The reader selectively toggled between the two images (returning to the first image as desired) and was forced to answer if they are distinguishable or not. Based on the reader’s response, the QUEST algorithm [31] calculated the CR for the next trial. The CR for the initial trial was 5:1. Details of the QUEST were described in elsewhere [31].

Prior to the formal visual analysis, the readers were instructed on how to perform the visual analysis with five example images which were not included in the test dataset. The readers also had a chance to perform the analysis with another three example image pairs by themselves so that they could become familiar with the visual analysis.

Although we selected images containing abnormalities, the readers were asked not to confine their visual analysis to the pathologies. Instead, the readers were asked to examine an entire image to find any image differences. When analyzing the abdomen CT images, the readers were asked to focus particularly on the small vessels and edges of the organs and the texture of solid organs and soft tissues. For the chest CT images, the readers were asked to focus on the small airways, pulmonary vessels, interlobular septa, interlobar fissures, and the texture of the pulmonary parenchyma.

### C. Selection of image features as independent variables

As inspired from the literature and our observations, we selected five image features of image standard deviation (*Image_SD*), image entropy (*Image_entropy*), relative percentage of low frequency energy (*Percentage_LF*), variation in high frequency (*Variation_HF*), and visual complexity (*Visual_complexity*) as the candidates for the determinants of the VLTs of CT images. Those five image features were measured for each image using Matlab (version 2011a, Mathworks, Nattick, Mass). The detail of those image features is described elsewhere [25].

### D. Selection of DICOM header information as independent variables

We selected four DICOM tags which were considered to affect the compressibility as the candidates for the independent variables based on the results of the previous study: [19] the effective tube current-time product (*effective mAs*; tag number: 0018, 9332); section thickness (*ST*; tag number: 0018, 0050); field of view (*FOV*; tag number: 0018, 0090), and reconstruction filter type (tag number: 0018, 9320).

### E. Construction of the VLT prediction model

To construct and validate a prediction model, we applied an analysis scheme widely used in the machine learning field [32]. The 206 images were divided randomly into two groups: 103 images for the training set and 103 images for the testing set. With the training set, an optimal subset of independent variables was determined using multiple linear regression (MLR) by performing backward stepwise selection using the likelihood-ratio statistic (*p* = 0.05 for entry and *p* = 0.10 for removal) as a selection criterion [32]. At each step in the backward stepwise selection, four-fold cross-validation scheme was used.

With the determined subset of independent variables, a final model was constructed by fitting the MLR on the entire training set while regarding the VLTs determined with median value of the radiologists’ responses (*VLT*_*rad*_) as dependent variable. The constructed MLR model was then validated using the testing set. The detail of the validation is described in the subsequent statistical analysis section.

The time for computing the VLTs predicted by the model (*VLT*_*model*_) was measured for each image. In this calculation, we included the time to load input files to the memory and to save output files to the storage. We used a PC platform running 64-bits Windows 7 (Microsoft Co., Redmond, WA) with a 3.2 GHz quad-core processor (i7-3930 k; Intel Co., Santa Clara, CA) and 32 GB main memory.

### F. Image fidelity metrics

To compare the performance of the prediction model with those of the pre-existing image fidelity metrics, we tested two metrics, peak signal-to-noise ratio (PSNR) [15] and high-dynamic range visual difference predictor (HDRVDP) [33]. These metrics have been widely tested in predicting radiologists’ perception of compression artifacts in CT images [5, 14–17, 34]. The detail of the metrics is described in Appendix.

Each of the two metrics takes two images (original and distorted images) as input and calculates the degree of the fidelity of the distorted image. Thereby, to calculate the VLT of a CT image using the metrics, it is necessary to iteratively compress and measure the image fidelity for multiple CRs until the measured fidelity reaches a cutoff value predefined by the metric.

The cutoff value of each of the metrics was determined using the training set as follows. First, each image on the training set was compressed to its *VLT*_*rad*_. Second, the metric value was calculated for each pair of the original image and compressed image to *VLT*_*rad*_. Note that if a metric is completely accurate in predicting the *VLT*_*rad*_, then the metric outputs for the *VLT*_*rad*_-compressed images should be a constant value. The cutoff value was defined as the mean of the metric values for the entire training set (*N* = 103) to minimize the deviation between the cutoff value and metric values.

The VLTs of each image using PSNR or HRDVDP (*VLT*_*(PSNR or HDRVDP)*_) on the testing set was calculated with the metrics’ own cutoff values using the iteration scheme. The times for computing the *VLT*_(*PSNR or HDRVDP*)_ were measured for each image.

### G. Statistical analysis

The sample size of 206 images (103 images for training set and 103 images for testing set) was determined to provide narrow two-sided 95% confidence intervals (CIs) for the absolute difference between VLT_rad_ and VLT_MLR_ (|VLT_rad-MLR_|) as follows. First, to measure the standard deviation (SD) of | VLT_rad-MLR_ |, we conducted a preliminary test with 100 images. The 100 images were repeatedly divided (200 times) randomly into two sets, a training (50 images) and testing (50 images) data sets. For each division, an MLR model was constructed on the training and |VLT_rad-MLR_| was measured on the testing set. The mean of SD of the 200 |VLT_rad-MLR_| was 0.51. With this SD, the sample size of a testing set was estimated to be 103 to construct a 95% CI of | VLT_rad-MLR_| with the width of no greater than 0.10. In addition to the testing set, a separate sample of the same size was required for the training set (*n* = 103).

Interobserver agreement between the five readers was evaluated by measuring the ICC coefficient. Using the testing set, the performance of each of the MLR model and the two metrics was evaluated by measuring ICC coefficient, and bland-Altman plot between *VLT*_*radiologist*_, and *VLT*_(*MLR* model, *PSNR, or HDRVDP*)_. The difference between |*VLT*_*rad-model*_|, |*VLT*_*rad-PSNR*_|, and |*VLT*_*rad-HDRVDP*_| were compared using the repeated measures analysis of variance (ANOVA) with a post-hoc analysis. The time for computing VLTs between MLR model, PSNR, and HDRVDP were compared using the repeated ANOVA with a post-hoc analysis. *P* < 0.05 was considered to indicate a statistically significant difference.

## Results

### A. VLT measured by radiologists

**Fig. 2:**
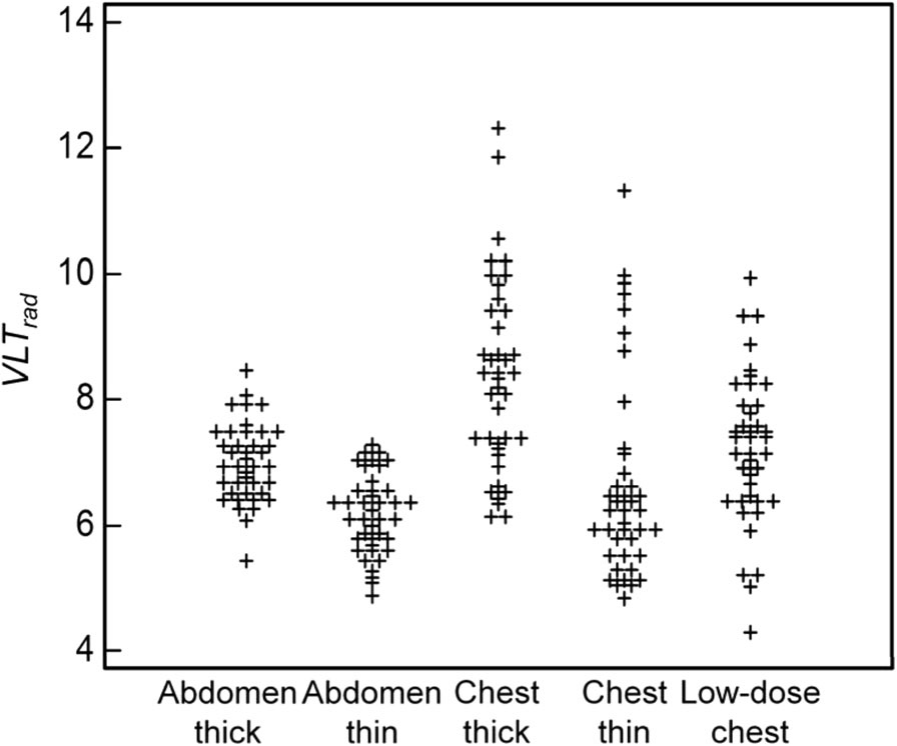
Scatter plot of the radiologists’ pooled responses. The *VLT*_*rad*_ represents the visually lossless thresholds determined with median value of the five radiologists’ responses for each image

The ICC coefficient between the five readers was 0.56 (95% CI, 0.53, 0.57; *p* < 0.01). The VLTs varied with different subsets, especially different section thicknesses (Fig. 2). The *VLT*_*rad*_ of thick sections were significantly higher than those of thin sections for both of abdomen (thick vs. thin, 7.3 ± 0.5 vs. 6.3 ± 0.4; *p* < 0.01) and chest (9.2 ± 1.1 vs. 7.1 ± 1.3; *p* < 0.01). The VLTs of chest thick sections were significantly higher than those of low-dose chest images (9.2 ± 1.1 vs. 7.5 ± 1.4; *p* < 0.01).

### B. Optimal variable selection

According to the results of backward stepwise selection, the *ST*, *effective mAs*, and *reconstruction filter* among the DICOM tags and *Visual_complexity*, and *Variation_HF* among the image features were determined as the optimal subset of independent variables while the remaining variables were excluded.

### C. Performance of MLR model, PSNR, and HDR-VDP (Figs. 3 and 4)

The means of |*VLT*_*rad-MLR*_|, |*VLT*_*rad-PSNR*_|, and |*VLT*_*rad-HDRVDP*_| were 0.58 (95% CI, 0.48, 0.67), 0.73 (0.61, 0.85), and 0.68 (0.58, 0.79), respectively, showing significant difference between them (*p* < 0.01). According to the post-hoc analysis, the significant difference was shown between |*VLT*_*rad-MLR*_| and |*VLT*_*rad-PSNR*_|. The ICC coefficients of MLR model, PSNR, and HDRVDP were 0.88 (95% CI, 0.81, 0.95), 0.84 (0.77, 0.91), and 0.85 (0.79, 0.91). The Bland-Altman plot demonstrates the discrepancy between *VLT*_*rad*_ and *VLT*_(*MLR model, PSNR, or HDRVDP*)_ with a mean difference (bias) and a 95% confidence limit of agreement (Fig. 5).

**Fig. 3:**
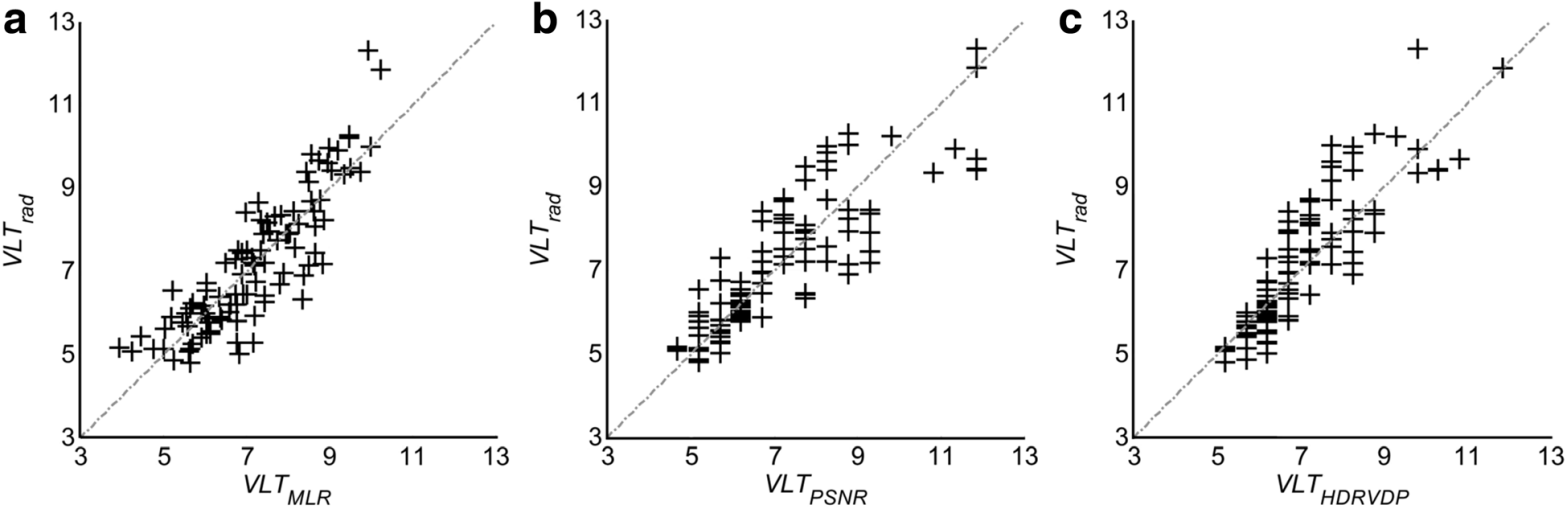
Scatter plots of *VLT*_*rad*_ and *VLT*_(*MLR model, PSNR or HDRVDP*)_ for the MLR model (**a**), for the PSNR (**b**) and for the HDRVDP (**c**). Symbol + represents each image

**Fig. 4:**
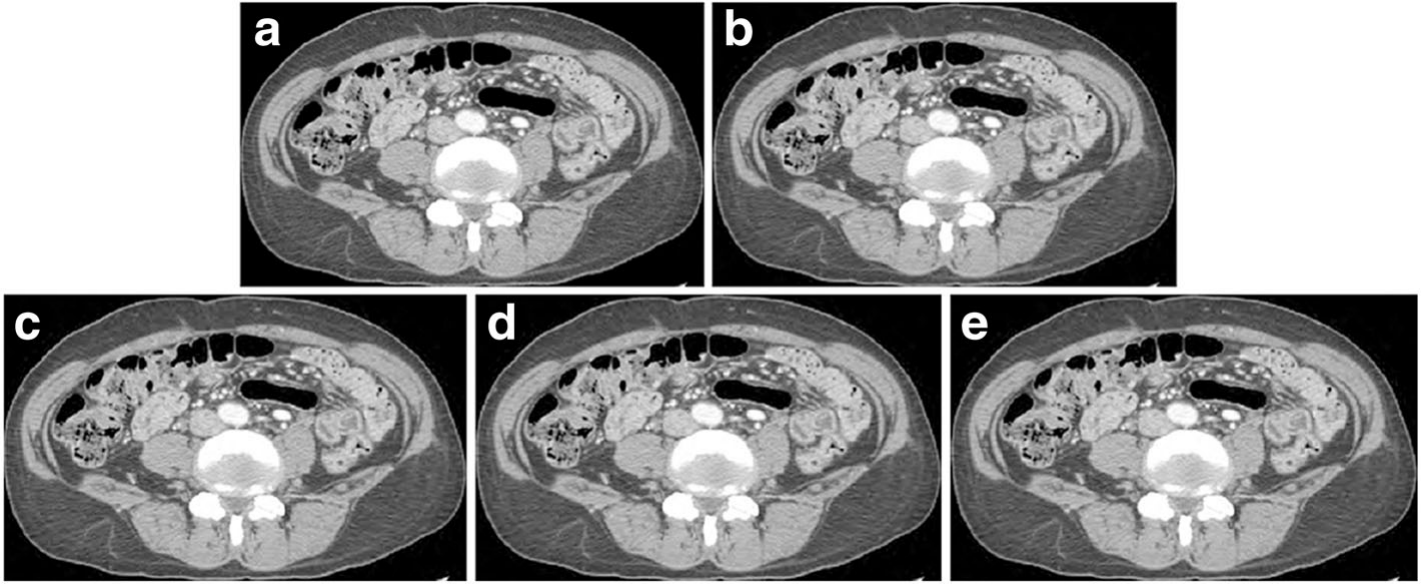
JPEG 2000 compressed CT images in transverse abdominal view of a 78-year-old female. (**a**) the original image. (**b**) the compressed image by a radiologist. (compression ratio 8.6:1.) The compressed images using (**c**) the MLR model (8.8,1), (**d**) the PSNR (9.5,1), and (**e**) the HDRVDP (9.3,1) respectively. Window width and level are set to 400 and 20 HU. Since the images are generated by visually lossless compression methods, all the images are indistinguishable from the original image

**Fig. 5:**
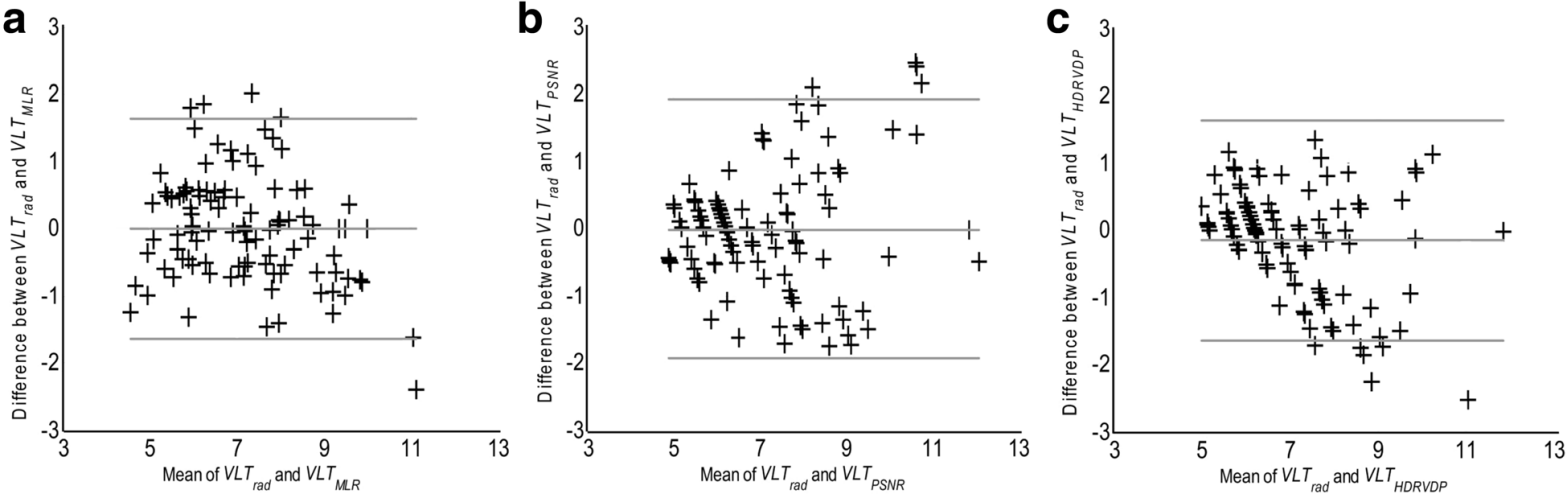
Bland-Altman plots between *VLT*_*rad*_ and *VLT*_*(MLR model, PSNR, or HDRVDP)*_ for the MLR model (**a**), for the PSNR (**b**) and for the HDRVDP (**c**). Symbol + represents each image

### D. Computing time of image features

The mean computing times for calculating VLT per image were 1.5 ± 0.1 s, 3.9 ± 0.3 s, and 68.2 ± 1.4 s, for MLR metric, PSNR, and HDRVDP, respectively. The differences between them were significant (*p* < 0.01).

## Discussion

In this study, we proposed a MLR model which predicts the VLT of JPEG2000 compressed CT images using the image features and DICOM header information. The mean of absolute difference between the VLTs measured by radiologists and the VLTs calculated by the MLR model was 0.58. The ICC coefficient of the VLTs measured by radiologists and the VLTs calculated by the MLR model was 0.88. The proposed model showed superior or comparable performance to those of the PSNR and HDRVDP while requiring less computational expenses. Our model utilized “*visually lossless*” compression, and does not interfere with a doctor’s diagnosis. Human visual system cannot find the difference between the original image and the compressed image resulting from the model. In clinical applications, it is crucial to compute the optimal VLT value to keep the quality of the compressed image that does not degrade the diagnostic value in any case. Therefore, the proposed model would be promising to be used for adaptive compression of CT images.

According to our results, among the DICOM header tags, the *ST*, *effective mAs*, and *reconstruction filter* played an important role in predicting the VLT of CT images. These results were expected since those DICOM tags are theoretically related to image noise and thus to the degree of compression artifacts. In addition, the image features of *Variation_HF* and *Visual_complexity* are also shown as factors predicting the VLT of CT images, which are devised by being inspired from the previous research on the modeling of human visual system (HVS) [25]. Note that there might exist other HVS-related image features which outperform those two image features.

Previous studies [5, 14–18] evaluated several image fidelity metrics, such as PSNR and HDRVDP in measuring the fidelity of compressed CT images. The principal goal of these studies was to introduce the image fidelity metrics into an adaptive compression. However, those metrics have a couple of limitations from a practical viewpoint. First, they analyze the image characteristics of a compressed image in comparison to its original, requiring a substantial computational expense. Second, to achieve an optimal compression level for an image, it is necessary to iteratively compress the image to multiple compression levels and to measure the image fidelity at each compression level until the measured fidelity reaches a predefined threshold. In contrast, the prediction model proposed in this study can directly predict VLTs of CT images without the iterative compression and fidelity measurement at multiple CRs, thereby computationally efficient. In an academic medical center with 900-bed tertiary care, where this study is conducted, the total number of CT examinations from August 2011 to January 2012 is 43,854. The required time and computational power for compressing all the CT examinations in such a great scale is not trivial. Using inefficient compression models may increase the waiting time of patients, particularly for the model like HDRVDP which requires about 46 times more computational cost comparing to our method. Furthermore, the models can probably induce unnecessary load on picture archiving and communication system (PACS) and gradually degenerate durability of the system.

Our model cannot be directly applied to clinical practice due to the following reasons: first, this study used a small number of test images; second, we did not test all available DICOM header information and image features. Furthermore, more sophisticated classification methods exist as an alternative to MLR, such as artificial neural network, support vector machine, and AdaBoost [32]. However, it should be noted that our study did not intend to suggest a prediction model which can serve as a global compression guideline for all CT images. Instead, the purpose of our study is rather to propose a new scheme of adaptive compression which directly predicts the VLT of a given CT image solely using the original image without compression.

## Conclusion

In conclusion, we proposed a MLR model which directly predicts the VLT of a given CT image solely using the original image without compression. The proposed model showed superior or comparable performance to those of image fidelity metrics while requiring less computational expenses. The model would be promising to be used for adaptive compression of CT images.

## Appendix

### Description of image fidelity metrics

The PSNR and HDRVDP were performed after converting the 12-bit images to 8-bit images by applying the same window settings used in the human visual analysis. This conversion was to reproduce the clinical practice, in which images are displayed with an appropriate window setting.

### PSNR

The PSNR [15] has been widely used in measuring the degree of image distortion due to its computational simplicity. The PSNR (in decibels [dB]) was calculated as follows:

1 $$ PSNR=20{\log}_{10}\left(\frac{255}{RMSE}\right), $$

Where

2 $$ RMSE=\sqrt{\frac{\sum \limits_{x=1}^{512}\sum \limits_{y=1}^{512}{\left(f\left(x,y\right)-g\left(x,y\right)\right)}^2}{512^2}}, $$

and where RMSE stands for root-mean-square error and *f*(*x*, *y*) and *g*(*x*, *y*) are the pixel values in the original and compressed images, respectively.

### HDRVDP

The HDRVDP, which is publicly available [33, 35], is an extension of Visual Difference Predictor [36], one of the most widely used perceptual metrics simulating individual components of the HVS. Because modern medical display systems are significantly brighter than general-purpose displays, the HDRVDP, which covers a wide range of luminance, is likely suitable for medical applications. The HDRVDP has been reported to accurately reproduce radiologists’ perception of compression artifacts in CT images [5, 14–17, 37].

The HDRVDP takes two images as input and then outputs a probability-of-detection map in which the pixel value indicates the probability, ranging from 0 to 1, that an observer viewing the two images will detect the difference at that pixel location. The map was summarized into a single value representing the overall perceptual image fidelity using the Minkowski metric [38] as follows:

3 $$ HDR- VDP={\left(\sum \limits_u\sum \limits_v\left\{p{\left(u,v\right)}^{\beta}\right\}\right)}^{\frac{1}{\beta }}, $$

where *p*(*u*, *v*) is the probability-of-detection map. *β* was set to 2.4 according to the results of previous studies [18].

## Abbreviations

ANOVA: Analysis of variance
CI: Confidence interval
CR: Compression ratio
CT: Computed tomography
DICOM: Digital imaging and communications in medicine
FOV: Field of view
HDRVDP: High-dynamic range visual difference predictor
HVS: Human visual system
ICC: Intra-class correlation
JPEG2000: Joint photographic experts group 2000
MLR: Multiple linear regression
PACS: Picture archiving and communication system
PSNR: Peak signal-to-noise ratio
SD: Standard deviation
ST: Section thickness
VLT: Visually lossless threshold

## References

[1] Koff D, Bak P, Brownrigg P, Hosseinzadeh D, Khademi A, Kiss A, Lepanto L, Michalak T, Shulman H, Volkening A (2009). Pan-Canadian evaluation of irreversible compression ratios ("Lossy" compression) for development of national guidelines. J Digit Imaging.

[2] Lee KH, Kim YH, Kim BH, Kim KJ, Kim TJ, Kim HJ, Hahn S (2007). Irreversible JPEG 2000 compression of abdominal CT for primary interpretation: assessment of visually lossless threshold. Eur Radiol.

[3] Lee KH, Lee HJ, Kim JH, Kang HS, Lee KW, Hong H, Chin HJ, Ha KS (2005). Managing the CT data explosion: initial experiences of archiving volumetric datasets in a mini-PACS. J Digit Imaging.

[4] Rubin GD (2000). Data explosion: the challenge of multidetector-row CT. Eur J Radiol.

[5] Kim KJ, Kim B, Lee KH, Mantiuk R, Kang HS, Seo J, Kim SY, Kim YH (2009). Objective index of image fidelity for JPEG2000 compressed body CT images. Med Phys.

[6] Cosman PC, Davidson HC, Bergin CJ, Tseng CW, Moses LE, Riskin EA, Olshen RA, Gray RM (1994). Thoracic CT Images: effect of lossy image compression on diagnostic accuracy. Radiology.

[7] Goldberg MA, Gazelle GS, Boland GW, Hahn PF, Mayo-Smith WW, Pivovarov M, Halpern EF, Wittenberg J (1997). Focal hepatic lesions: effect of three-dimensional wavelet compression on detection at CT. Radiology.

[8] Ko JP, Chang J, Bomsztyk E, Babb JS, Naidich DP, Rusinek H (2005). Effect of CT image compression on computer-assisted lung nodule volume measurement. Radiology.

[9] Ko JP, Rusinek H, Naidich DP, McGuinness G, Rubinowitz AN, Leitman BS, Martino JM (2003). Wavelet compression of low-dose chest CT data: effect on lung nodule detection. Radiology.

[10] Ohgiya Y, Gokan T, Nobusawa H, Hirose M, Seino N, Fujisawa H, Baba M, Nagai K, Tanno K, Takeyama N, Munechika H (2003). Acute cerebral infarction: effect of JPEG compression on detection at CT. Radiology.

[11] Zalis ME, Hahn PF, Arellano RS, Gazelle GS, Mueller PR (2001). CT colonography with teleradiology: effect of lossy wavelet compression on polyp detection-initial observations. Radiology.

[12] Bajpai V, Lee KH, Kim B, Kim KJ, Kim TJ, Kim YH, Kang HS (2008). The difference of compression artifacts between thin- and thick-section lung CT lmages. Am J Roentgenol.

[13] Woo HS, Kim KJ, Kim TJ, Hahn S, Kim BH, Kim YH, Yoon CJ, Lee KH (2007). JPEG 2000 compression of abdominal CT: difference in compression tolerance between thin- and thick-section images. Am J Roentgenol.

[14] Kim B, Lee KH, Kim KJ, Mantiuk R, Bajpai V, Kim TJ, Kim YH, Yoon CJ, Hahn S (2008). Prediction of perceptible artifacts in JPEG2000 compressed abdomen CT images using a perceptual image quality metric. Acad Radiol.

[15] Kim B, Lee KH, Kim KJ, Mantiuk R, Hahn S, Kim TJ, Kim YH (2008). Prediction of perceptible artifacts in JPEG2000 compressed chest CT images using mathematical and perceptual quality metrics. Am J Roentgenol.

[16] Kim B, Lee KH, Kim KJ, Mantiuk R, Kim HR, Kim YH (2008). Artifacts in slab average-intensity-projection images reformatted from JPEG 2000 compressed thin-section abdominal CT data sets. Am J Roentgenol.

[17] Kim KJ, Kim B, Lee KH, Kim TJ, Mantiuk R, Kang HS, Kim YH (2008). Regional difference in compression artifacts in low-dose chest CT images: effects of mathematical and perceptual factors. Am J Roentgenol.

[18] Kim KJ, Kim B, Mantiuk R, Richter T, Lee H, Kang HS, Seo J, Lee KH (2010). A comparison of three image fidelity metrics of different computational principles for JPEG2000 compressed abdomen CT images. IEEE Trans Med Imaging.

[19] Kim KJ, Kim B, Lee H, Choi H, Jeon JJ, Ahn JH, Lee KH (2011). Predicting the fidelity of JPEG2000 compressed CT images using DICOM header information. Med Phys.

[20] Digital Imaging and Communications in Medicine (DICOM). Part 14: gray scale standard display function. medical.nema.org/dicom/2004/04_14pu.pdf. Accessed 1 June 2012.

[21] Clunie DA, Mitchell PJ, Howieson J, Roman-Goldstein S, Szumowski J (1995). Detection of discrete white matter lesions after irreversible compression of MR images. AJNR Am J Neuroradiol.

[22] Erickson BJ, Manduca A, Palisson P, Persons KR, Earnest FT, Savcenko V, Hangiandreou NJ (1998). Wavelet compression of medical images. Radiology.

[23] Fidler A, Skaleric U, Likar B (2006). The impact of image information on compressibility and degradation in medical image compression. Med Phys.

[24] Janhom A, van der Stelt P, van Ginkel F (2000). Interaction between noise and file compression and its effect on the recognition of caries in digital imaging. Dentomaxillofac Radiol.

[25] Kim KJ, Kim B, Lee KH, Mantiuk R, Richter T, Kang HS. Use of image features in predicting visually lossless thresholds of JPEG2000 compressed body CT images: initial trial. Radiology. 2013; in press 10.1148/radiol.13122015 23630311

[26] Kim TJ, Lee KW, Kim B, Kim KJ, Chun EJ, Bajpai V, Kim YH, Hahn S, Lee KH (2008). Regional variance of visually lossless threshold in compressed chest CT images: lung versus mediastinum and chest wall. Eur J Radiol.

[27] Antonini M, Barlaud M, Mathieu P, Daubechies I (1992). Image coding using wavelet transform. IEEE Trans Image Processing.

[28] Zeng W, Daly S (2002). An overview of the visual optimization tools in JPEG 2000. Signal Process: Image Comm.

[29] Liu Z, Karam LJ, Watson AB (2006). JPEG2000 encoding with perceptual distortion control. IEEE Trans Image Processing.

[30] Tan D, Tan C, Wu H (2010). Perceptual color image coding with JPEG2000. IEEE Trans Image Processing.

[31] Macmillan NA (2001). Threshold estimation: the state of the art. Attention, Perception, Psychophysics.

[32] Friedman J, Hastie T, Tibshirani R. The elements of statistical learning. New York: Springer series in statistics; 2001.

[33] R. Mantiuk, HDR visual difference predictor. http://sourceforge.net/projects/hdrvdp. Accessed 4 July 2006.

[34] Shannon CE (1948). A mathematical theory of communication. Bell Syst Tech J.

[35] Mantiuk R, Daly S, Myszkowski K, Seidel H-P (2005). Presented at the proc human vision and electronic imaging X, IS&T/SPIE's 17th annual symposium on electronic imaging.

[36] Daly S, Watson AB (1993). The visible differences predictor: an algorithm for the assessment of image fidelity. Digital images and human vision.

[37] Kim B, Lee H, Kim KJ, Seo J, Park S, Shin YG, Kim SH, Lee KH (2011). Comparison of three image comparison methods for the visual assessment of the image fidelity of compressed computed tomography images. Med Phys.

[38] Quick RF (1974). A vector-magnitude model of contrast detection. Biol Cybern.

[39] G. Bongartz, S. J. Golding, A. G. Jurik, M. Leonardi, E. van Persijn van Meerten, R. Rodríguez, K. Schneider, A. Calzado, J. Geleijns, K. A. Jessen, W. Panzer, P. C. Shrimpton and G. Tosi, Bongartz G, Golding SJ, Jurik AG, et al. European guidelines for multislice computed tomography. http://www.drs.dk/guidelines/ct/quality/index.htm. Accessed 1 June 2012.

